# Regulation of the V-ATPase along the Endocytic Pathway Occurs through Reversible Subunit Association and Membrane Localization

**DOI:** 10.1371/journal.pone.0002758

**Published:** 2008-07-23

**Authors:** Céline Lafourcade, Komla Sobo, Sylvie Kieffer-Jaquinod, Jérome Garin, F. Gisou van der Goot

**Affiliations:** 1 Global Health Institute, Ecole Polytechnique Fédérale de Lausanne, Faculty of Life Sciences, Lausanne, Switzerland; 2 CEA, DSV, iRTSV, Laboratoire d'Etude de la Dynamique des Protéomes, INSERM U880, Grenoble, France; Université de Toulouse, France

## Abstract

The lumen of endosomal organelles becomes increasingly acidic when going from the cell surface to lysosomes. Luminal pH thereby regulates important processes such as the release of internalized ligands from their receptor or the activation of lysosomal enzymes. The main player in endosomal acidification is the vacuolar ATPase (V-ATPase), a multi-subunit transmembrane complex that pumps protons from the cytoplasm to the lumen of organelles, or to the outside of the cell. The active V-ATPase is composed of two multi-subunit domains, the transmembrane V_0_ and the cytoplasmic V_1_. Here we found that the ratio of membrane associated V_1_/Vo varies along the endocytic pathway, the relative abundance of V_1_ being higher on late endosomes than on early endosomes, providing an explanation for the higher acidity of late endosomes. We also found that all membrane-bound V-ATPase subunits were associated with detergent resistant membranes (DRM) isolated from late endosomes, raising the possibility that association with lipid-raft like domains also plays a role in regulating the activity of the proton pump. In support of this, we found that treatment of cells with U18666A, a drug that leads to the accumulation of cholesterol in late endosomes, affected acidification of late endosome. Altogether our findings indicate that the activity of the vATPase in the endocytic pathway is regulated both by reversible association/dissociation and the interaction with specific lipid environments.

## Introduction

During evolution, compartmentalization of the intracellular space has allowed to spatially restrict and optimize certain biochemical reactions and pathways. Therefore the lumens of different organelles have different properties in terms of ion concentrations, redox states and also pH. Organellar pH is tightly regulated ranging from neutral in the endoplasmic reticulum, to mildly acidic in early endosomes and highly acidic in late endosomes/lysosomes [1]. Acidic pH affects a number of biological events such as membrane trafficking, dissociation of ligand-receptor complexes after internalization and activation of lysosomal enzymes [1]. Although pH regulation can be modulated by a variety of factors such as proton leak, ClC chloride channels or Na,K-ATPases [2], [3], [4], [5], it is primarily determined by the activity of the vacuolar ATPase (V-ATPase), which is expressed in all eukaryotes from yeast to mammals [6], [7].

The V-ATPase is a multi-subunit complex composed of two domains, a peripheral V_1_ domain containing the ATPase activity and a membrane bound V_0_ domain responsible for translocation of protons across the membrane [6], [8]. The central function of the V-ATPase is to pump protons from the cytoplasm to the lumen of organelles. In some specialized cells, the V-ATPase can also be found at the plasma membrane. Its role is then either to acidify the extracellular medium such as around osteoclasts and renal cells [9], [10], [11], or to control the cytoplasmic pH as in neutrophiles and macrophages [12], [13]. The proton pump also appears to be involved in cancer through the promotion of metastasis and tumor progression and it is therefore considered as a potential drug target [6], [13].

Despite the importance of the V-ATPase in physiological and pathological processes, the exact mechanisms that control the activity of the V-ATPase remain to be fully elucidated. Four regulatory mechanisms have been described to date. The first is the reversible dissociation of the catalytic V_1_ domain from the membrane-associated V_0,_ which was observed in *S. cerevisiae* and *Manduca sexta* upon glucose deprivation and starvation respectively [14], [15]. Change in V_1_/V_0_ association was also reported during maturation of murine dendritic cells [16]. The second mechanism involves the abundance of the proton pump at a given site. In cells such as renal cells and osteoclasts, where the proton pump is at the cell surface, acid secretion was indeed found to be modulated by a differential surface expression of the V-ATPase through reversible exocytosis and endocytosis of the pump [17]. The third mechanism by which the activity of the V-ATPase could be modulated is by changing of the coupling efficiency between ATP hydrolysis and proton translocation, attributable to different isoforms of subunit V_0_a. This differential coupling efficiency was proposed to explain that lysosomes are more acidic than the Golgi [1], [6], [18] [19]. Finally, it has been proposed that specific lipids can affect the activity of the V-ATPase. More specifically it was shown in yeast that sphingolipids with a C26 acyl group are required for generating V1 domains with ATPase activity [20]. Interestingly, several V-ATPase inhibitors where shown to incorporate into the lipid bilayer and affect the V-ATPase structural flexibility [21], [22].

In this study, our interest was to get a better understanding of how pH is controlled along the endocytic pathway in mammalian cells, since most studies on V-ATPase regulation where performed in yeast. We found that the increased acidity of late endosomes is not due to a higher density of proton pumps but rather to an increase in the V_1_/V_0_ ratio when compared to early endosomes. Thus regulation would occur via reversible association/dissociation, reminiscent of what occurs found in yeast. Additionally, we found that the lipid environment of the V-ATPase is of essential importance for its activity, suggesting a second mode of regulation and highlighting that the V-ATPase activity is modulated by multiple, simultaneously operating, mechanisms.

## Results

### DRM association of all the subunits of the V-ATPase

Endosomes, and in particular late endosomes, are well known to be composed of mosaics of domains [23], [24]. We have shown that late endosomes, in particular, contain raft-like domains that are rich in cholesterol and sphingomyelin and resistant to extraction in non-ionic detergents at 4°C [25], [26]. To further characterize late endosomal DRMs, we have here performed a proteomic analysis. Proteomics analysis of DRM fractions have been reported in numerous studies [27], [28], [29], [30], [31], but this is the first that proteomics was performed on DRMs of a purified endocytic organelle. As previously, late endosomes were obtained from Baby Hamster Kidney cells, since a well-established subcellular fractionation protocol to purify late endosomes is available for this cell line [25], [32]. We have previously shown using surface biotinylation of proteins that this fraction does not contain detectable amounts of plasma membrane [33]. Moreover this late endosomal fraction is not contaminated by Golgi, endoplasmic reticulum, early endosomal or caveolar membranes, as shown by western blotting using marker proteins [34]. It is likely that the high purity of this fraction is due to the fact that the late endosomal fraction has a very low buoyancy and thus migrates through the entire gradient to the top of the tube, far away from all other cellular compartments. The llate endosomal fraction was subsequently submitted to solubilization in triton X-100 at 4°C and the Detergent-Resistant-Membranes (DRMs, fractions 1 and 2 from the top of the gradient) were separated from the Detergent-Soluble-Membranes (DSMs, fractions 5 and 6) using floatation gradients. The proteomes of both the DRMs and DSMs were determined by mass spectrometry after appropriate trypsinization and sample preparation.

Some 126 and 161 proteins were identified in the DRM and DSM fractions respectively, 44 of which were found in both fractions (Table 1 and 2), which jointly provide a global proteome of the organelle. Reassuringly, known late endosome specific proteins such as the small GTPase Rab7, the lysosomal glycoprotein LAMP2, or the NPC1 protein involved in Niemann Pick type C disease [35] were found. Also the DRM fraction contained most of the well-documented rafts markers such as caveolin-1 and 2 –involved in the formation of caveolae, in signaling and in lipid regulation [36]–, erlin 2, –a DRM associated ER protein [37], flotillins 1 and 2 [38], which were also found in the DSM in agreement with detectable amounts by western blotting [25], stomatin and the hyaluronic acid receptor CD44 [39].

**Table 1:** Proteins identified in the late endosomal DRM fraction.

Numb	Uniprot Numb	Protein name	Protein family	Mascot score	Mw	Cov (%)	Nb pep
1	P56564	Excitatory amino acid transporter 1	AA transport	150.46	59584	7.21	2
2	**Q3UFR4**	**Neutral amino acid transporter ASCT2**	AA transport	186.95	58327	6.42	3
3	Q9JKY1	Peroxiredoxin-1	Cell redox homeostasis	45.56	22248	5.45	1
4	**Q71FK5**	**Actin, cytoplasmic 1 (Beta-actin)**	Cytoskeleton	1034.51	41710	54.35	15
5	P63260	Actin, cytoplasmic 2 (Gamma-actin)	Cytoskeleton	1071.66	41766	54.35	15
6	Q7TSH1	Actr3b protein	Cytoskeleton	55.1	48539	2.49	1
7	Q8BMK4	Cytoskeleton-associated protein 4	Cytoskeleton	74.92	60655	2.9	1
8	P47757	F-actin-capping protein subunit beta	Cytoskeleton	44.23	31195	3.53	1
9	Q5M810	Myo1g protein	Cytoskeleton	114.37	58945	7.1	2
10	Q3UFT0	myosin heavy chain IX	Cytoskeleton	108.31	110908	4.27	2
11	Q63355	Myosin I heavy chain	Cytoskeleton	1530.65	118017	27.33	23
12	P14869	Myosin regulatory light chain 2-A	Cytoskeleton	80.24	34195	3.87	1
13	P46735	Myosin-Ib	Cytoskeleton	45.16	128447	0.77	1
14	Q9ERB6	Nuclear myosin I beta	Cytoskeleton	1508.15	119802	27.09	23
15	P30427	Plectin-1	Cytoskeleton	42.29	533214	0.23	1
16	Q9EPK2	Protein XRP2	Cytoskeleton	39.92	39220	3.93	1
17	**P54116**	**Stomatin**	Cytoskeleton	192.32	31384	15.79	3
18	P68372	Tubulin beta-2C chain	Cytoskeleton	144.93	49799	6.42	3
19	Q3TWV0	vimentin	Cytoskeleton	97.46	53633	4.72	2
20	**Q8K3H8**	**Calnexin**	ER chaperone	48.48	67422	2.61	1
21	Q8BFZ9	Erlin-2 precursor	ERAD pathway	129.07	37849	10.76	3
22	**O08917**	**Flotillin-1**	Flotillin complex	900.61	47484	42.92	14
23	**Q9Z2S9**	**Flotillin-2**	Flotillin complex	782.94	47009	34.43	12
24	**P17809**	**Glucose transporter type 1 (GLUT-1)**	Glucose transporter	105.89	53899	3.68	2
25	Q3UAZ6	ATP-binding cassette, sub-family B (MDR/TAP), member 6	Heme homeostasis	41.06	84765	1.95	1
26	O88783	Coagulation factor V	Ion binding	43.16	247076	0.45	1
27	Q6PFA8	Moxd1 protein	Ion binding	89.33	69734	3.63	2
28	Q3TVE3	similar to S100 calcium-binding protein A16	Ion binding	49.01	14287	8.53	1
29	**Q7TMC7**	**Ab2-417 (Cc1-8)**	Ion transport	97.43	107343	3.08	2
30	P51881	ADP/ATP translocase 2	Ion transport	92.37	32779	7.74	2
31	**Q8VDN2**	**Na(+)/K(+) ATPase alpha-1 subunit**	Ion transport	634.88	112910	14.91	10
32	**Q3TX38**	**voltage-dependent anion channel 3**	Ion transport	143.97	30733	12.19	3
33	**Q9Z2L0**	**Voltage-dependent anion-selective channel protein 1 (VDAC-1)**	Ion transport	107.19	30606	7.55	2
34	**Q60930**	**Voltage-dependent anion-selective channel protein 2 (VDAC-2)**	Ion transport	49.07	31713	4.07	1
35	O08532	Voltage-dependent calcium channel subunit alpha-2/delta-1	Ion transport	508.19	124551	12.37	9
36	**P51909**	**Apolipoprotein D**	Lipid biogenesis	58.56	21596	6.12	1
37	Q9JKU9	Sigma 1 receptor beta variant	Lipid biogenesis	42.74	21589	6.63	1
38	**P00762**	**Anionic trypsin-1 precursor**	Peptidase	81.74	25943	8.51	1
39	**P07356**	**Annexin A2**	Phospholipid binding/modifying	339.59	38521	24	6
40	Q8BG11	cadherin 13	Protein binding	43.41	78137	2.39	1
41	Q8BFU4	GAIP/RGS19 short isoform	Protein binding	48.57	22240	6.44	1
42	**Q4FCR4**	**Intercellular adhesion molecule 1**	Protein binding	72.74	53809	2.86	1
43	P70490	Lactadherin precursor	Protein binding	43.23	47382	2.56	1
44	Q6DI58	Rpl12 protein	Protein binding	53.03	22973	7.21	1
45	Q6PDW1	40S ribosomal protein S12	Protein biosynthesis	108.17	14505	16.79	1
46	**P62629**	**Elongation factor 1-alpha 1**	Protein biosynthesis	50.37	50082	5.27	1
47	Q3U561	ribosomal protein L10A	Protein biosynthesis	70.68	24800	5.78	1
48	Q3UBI6	ribosomal protein L7	Protein biosynthesis	53.16	31331	3.87	1
49	Q9QWC2	Ribosomal protein P2	Protein biosynthesis	40.29	5063	23.08	1
50	Q3UCL7	ribosomal protein S3	Protein biosynthesis	136.27	26658	16.12	3
51	**Q62186**	**Translocon-associated protein subunit delta precursor**	Protein biosynthesis	61.03	18924	6.4	1
52	Q3S4T7	78 kDa glucose-regulated protein precursor	Protein folding	268.69	72284	8.68	5
53	**P24369**	**Cyclophilin B**	Protein folding	38.8	22699	6.31	1
54	**P30412**	**Cyclophilin C**	Protein folding	253.35	22780	22.71	4
55	Q3KQJ4	Hspa8 protein (Fragment)	Protein folding	252.63	62117	8.87	5
56	Q6AYQ9	Peptidyl-prolyl cis-trans isomerase	Protein folding	211.16	22995	20.1	4
57	P62858	40S ribosomal protein S28	Protein processing	60.75	7836	16.9	1
58	Q9R0Y5	Adenylate kinase isoenzyme 1	Protein processing	51.75	21526	6.67	1
59	Q9WU83	Dolichol-phosphate mannosyltransferase	Protein processing	42.88	29636	4.09	1
60	**P57716**	**Nicastrin precursor**	Protein processing	43.83	78441	2.24	1
61	**Q6AXQ3**	**Viral oncogene yes-1 homolog 1**	Protein processing	249.57	54236	14	5
62	P25286	V0 subunit a1	Proton transport	419.99	96265	9.03	7
63	Q5XK06	V0 subunit C	Proton transport	45.38	20434	9.73	1
64	P51863	V0 subunit d	Proton transport	210.04	40275	7.92	3
65	Q3U5W3	V1 subunit A, isoform 1	Proton transport	790.08	68211	30.97	13
66	P62814	V1 subunit B2 (brain isoform)	Proton transport	639.65	56515	29.82	12
67	Q9Z1G3	V1 subunit C	Proton transport	176.41	43702	10.08	3
68	P57746	V1 subunit D	Proton transport	47.54	28351	6.23	1
69	Q3UK59	V1 subunit E isoform 1	Proton transport	357.23	26145	27	5
70	Q9D1K2	V1 subunit F	Proton transport	91.08	13362	17.36	2
71	Q8R2H0	V1 subunit G2	Proton transport	165.4	13659	12.9	2
72	Q8BVE3	V1 subunit H	Proton transport	38.7	55819	3.16	1
73	P63001	Ras-related C3 botulinum toxin substrate 1	Ras-related protein	345.83	21436	32.47	6
74	Q99JI6	Ras-related protein Rap-1b precursor	Ras-related protein	291.41	20812	32.28	5
75	**P61226**	**Ras-related protein Rap-2b precursor**	Ras-related protein	142.64	20491	20.43	2
76	**P62071**	**Ras-related protein R-Ras2 precursor**	Ras-related protein	125.1	23385	13.21	2
77	Q60522	CD44 antigen precursor	Receptor	127.99	46778	5.41	2
78	**P17852**	**Integrin alpha-3 precursor**	Receptor	150.39	118475	2.79	2
79	Q9ERD4	Ankyrin repeat-rich membrane-spanning protein	Signalling	45.27	190414	1.21	1
80	P32261	Antithrombin-III precursor (ATIII)	Signalling	68.51	51971	2.54	1
81	Q7TMA5	Apolipoprotein B-100	Signalling	276.81	535688	1.36	5
82	**P61022**	**Calcium-binding protein p22**	Signalling	93.05	22287	13.86	2
83	**P62204**	**Calmodulin**	Signalling	331.96	16696	54.97	4
84	Q9DAS9	G protein G(I)/G(S)/G(O) subunit gamma-12 precursor	Signalling	40.48	7861	22.54	1
85	**P62874**	**G protein G(I)/G(S)/G(T) subunit beta-1**	Signalling	195.17	37222	10.06	2
86	**P62880**	**G protein G(I)/G(S)/G(T) subunit beta-2**	Signalling	171.96	37176	10.09	2
87	P38403	G protein G(k) subunit alpha (G(i) alpha-3)	Signalling	177.74	40447	11.44	3
88	P08752	Guanine nucleotide-binding protein G(i)	Signalling	264.43	40314	14.21	4
89	Q6R0H7	Guanine nucleotide-binding protein G(s) subunit alpha isoforms	Signalling	206.68	121429	3.26	4
90	Q3U2W7	Kirsten rat sarcoma oncogene 2	Signalling	67.25	21400	6.19	1
91	**P62977**	**Ubiquitin**	Signalling	112.42	8560	32.47	2
92	Q6IN24	Galectin 8	sugar binding	96.51	36050	5.81	2
93	**Q9CQW2**	**ADP-ribosylation factor-like protein 8B**	Trafficking	77.66	21525	19.49	2
94	P17426	AP-2 complex subunit alpha-1	Trafficking	110.59	107596	2.45	2
95	Q9DBG3	AP-2 complex subunit beta-1	Trafficking	92.4	104516	4.95	2
96	P62743	AP-2 complex subunit sigma-1	Trafficking	50.54	17007	9.74	1
97	P49817	Caveolin-1	Trafficking	289.28	20525	40.32	6
98	Q8VIK7	Caveolin-2	Trafficking	46.57	18163	9.7	1
99	P08712	Endoplasmin	Trafficking	41.48	46765	3.29	1
100	Q6WRU0	GPI-anchored protein GREG	Trafficking	49.7	22812	5.31	1
101	Q5SW87	RAB1	Trafficking	144.33	15016	27.94	3
102	**Q4FJL0**	**RAB10**	Trafficking	129.62	22527	16.67	3
103	**Q91V41**	**RAB14**	Trafficking	51.95	23751	6.05	1
104	P35293	RAB18	Trafficking	48.64	23021	5.26	1
105	**P53994**	**RAB2A**	Trafficking	177.51	23533	20.19	3
106	**P61021**	**RAB5B**	Trafficking	36.86	23692	6.51	1
107	**Q3TCT9**	**RAB5C**	Trafficking	132.96	23397	21.23	3
108	**Q3U4W5**	**RAB6**	Trafficking	41.42	23531	5.63	1
109	**Q4AEF6**	**RAB7**	Trafficking	163.59	23489	17.37	3
110	P13596	RAB7a	Trafficking	140.11	94599	3.49	2
111	P63321	Ral-A precursor	Trafficking	121.59	23538	12.68	3
112	**Q3UZ06**	**SEC22 vesicle trafficking protein-like 1**	Trafficking	67.84	18935	6.98	1
113	O70377	SNAP-23	Trafficking	84.91	23220	18.48	2
114	Q5F234	Syntaxin 8	Trafficking	66.07	26908	7.38	1
115	**O35587**	**Transmembrane protein Tmp21 precursor**	Trafficking	90.42	24805	8.89	2
116	Q9WV55	VAMP-associated protein A (VAMP-A)	Trafficking	93.96	27262	5.67	2
117	O88384	Vesicle transport v-SNARE protein Vti1-like 1 (Vti1-rp1)	Trafficking	53.28	26697	4.96	1
118	**Q791V5**	**Mitochondrial carrier homolog 2**	Tranporter	52.55	33477	7.89	1
119	O54724	Polymerase I and transcript release factor	Transcription	138.19	43927	7.27	2
120	**Q9Z239**	**Phospholemman precursor**	Unknown cellular process	52.05	10316	12.9	1
121	Q3TH64	Q3TH64	Unknown cellular process	40.29	16236	23.13	1
122	Q6UL10	Q6UL10	Unknown cellular process	45.96	223986	0.59	1
123	Q9QUR8	Semaphorin-7A precursor	Unknown cellular process	60.59	74946	1.91	1
124	Q3TJK3	serine (or cysteine) proteinase inhibitor	Unknown cellular process	119.34	46505	9	2
125	**Q3TIP1**	**solute carrier family 3**	Unknown cellular process	40.75	10997	17.17	1
126	**Q6P791**	**UPF0404 protein C11orf59 homolog**	Unknown cellular process	137.22	17710	16.25	2

Mw: theoretical molecular weight (kDa). Cov (%), protein sequence coverage; Nb pep, number of peptides assigned. In bold are the proteins found in both DRM and DSM fractions.

**Table 2:** Proteins identified in the late endosomal DSM fraction.

Numb	Uniprot Numb	Protein name	Protein family	Mascot score	Mw	Cov (%)	Nb pep
1	Q60458	Cationic amino acid transporter-1	AA transport	97.13	35185	11.6	2
2	Q8VII6	Choline transporter-like protein 1	AA transport	47.02	73043	1.81	1
3	**Q3UFR4**	**Neutral amino acid transporter ASCT2**	AA transport	174.62	58327	6.42	3
4	P17897	Lysozyme C type P precursor	Bacteriolytic	49.06	16783	7.89	1
5	Q920L2	Succinate dehydrogenase [ubiquinone] flavoprotein subunit	Carbohydrate metabolism	55.31	71570	2.15	1
6	Q9Z0G9	Claudin-3	Cell adhesion	85.27	23269	13.27	2
7	Q3T1H3	Ncam1 protein	Cell adhesion	400.3	93515	11.65	6
8	Q91Z81	ERP57 protein	Cell redox homeostasis	493.44	56761	20.93	9
9	P20070	NADH-cytochrome b5 reductase 3	Cell redox homeostasis	243.98	34022	12.94	4
10	Q60451	NADPH-cytochrome P450 oxidoreductase	Cell redox homeostasis	46.89	75803	1.6	1
11	Q52KJ9	Thioredoxin domain containing 1	Cell redox homeostasis	120.38	31415	10.53	2
12	Q3THH1	thioredoxin domain containing 5	Cell redox homeostasis	326.36	48627	11.54	4
13	**Q71FK5**	**Actin, cytoplasmic 1 (Beta-actin)**	Cytoskeleton	583.76	41710	37.2	10
14	P18760	Cofilin-1	Cytoskeleton	111.27	18417	13.77	2
15	P47753	F-actin-capping protein subunit alpha-1	Cytoskeleton	54.64	32788	3.36	1
16	P62962	Profilin-1	Cytoskeleton	50.02	14816	10.45	1
17	**P54116**	**Stomatin**	Cytoskeleton	48.34	31384	4.21	1
18	P68361	Tubulin alpha-1B chain	Cytoskeleton	55.57	50120	1.98	1
19	P69893	Tubulin beta-5 chain	Cytoskeleton	115.73	49639	4.43	2
20	P02544	Vimentin	Cytoskeleton	305.99	53566	11.93	5
21	**Q8K3H8**	**Calnexin**	ER chaperone	119.25	67224	5.07	2
22	**O08917**	**Flotillin-1**	Flotillin complex	383.61	47484	20.19	6
23	**Q9Z2S9**	**Flotillin-2**	Flotillin complex	445.33	47009	20.84	7
24	Q9Z1W7	GP50	Folding and Transport regulator	264.19	46569	14.89	4
25	Q3TWF2	heat shock 70kD protein 5	Folding and Transport regulator	909.11	72419	27.66	15
26	P19378	Heat shock cognate 71 kDa protein	Folding and Transport regulator	500.69	70761	16.49	9
27	P35293	Heat shock protein HSP 90-beta	Folding and Transport regulator	44.7	23021	5.26	1
28	**P17809**	**Glucose transporter type 1 (GLUT-1)**	Glucose transporter	51.34	53899	1.64	1
29	P05064	Fructose-bisphosphate aldolase A	Glycolysis	64.07	39200	5.62	1
30	Q3THM2	glyceraldehyde-3-phosphate dehydrogenase	Glycolysis	91.15	35789	8.11	2
31	O70252	Heme oxygenase 2	Heme homeostasis	85.89	35716	12.35	2
32	Q1MWP8	EH-domain containing 4-KJR	Ion binding	38.62	61613	1.96	1
33	Q3U7R1	Extended-synaptotagmin-1 (E-Syt1)	Ion binding	114.58	121478	2.63	2
34	Q6PFA8	N-acylsphingosine amidohydrolase 1	Ion binding	45.67	69734	1.58	1
35	Q05186	Reticulocalbin-1 precursor	Ion binding	42.58	38090	4.34	1
36	**Q7TMC7**	**Ab2-417 (Cc1-8)**	Ion transport	110.61	107343	2.97	2
37	Q3V132	ADP/ATP translocase 4	Ion transport	47.67	35235	3.75	1
38	P15999	ATP synthase subunit alpha	Ion transport	58.99	59717	2.21	1
39	P56480	ATP synthase subunit beta	Ion transport	140.84	56265	5.09	2
40	O55143	endoplasmic reticulum calcium ATPase 2	Ion transport	107.98	114784	3.45	2
41	Q09143	High affinity cationic amino acid transporter 1	Ion transport	98.32	67048	3.94	2
42	Q91VS7	Microsomal glutathione S-transferase 1	Ion transport	83.61	17409	7.59	1
43	**Q8VDN2**	**Na(+)/K(+) ATPase alpha-1 subunit**	Ion transport	621.11	112910	16.37	10
44	P11505	Plasma membrane calcium-transporting ATPase 1	Ion transport	522.49	138632	10.32	11
45	**Q3TX38**	**voltage-dependent anion channel 3**	Ion transport	232.58	30733	19.35	4
46	**Q9Z2L0**	**Voltage-dependent anion-selective channel protein 1 (VDAC-1)**	Ion transport	245.29	30606	23.38	5
47	**Q60930**	**Voltage-dependent anion-selective channel protein 2 (VDAC-2)**	Ion transport	177.68	31713	14.93	3
48	**P51909**	**Apolipoprotein D**	Lipid biogenesis	62.59	21596	6.12	1
49	Q8BLF1	Arylacetamide deacetylase-like 1	Lipid biogenesis	122.47	45711	5.06	2
50	O88531	Palmitoyl-protein thioesterase 1 precursor	Lipid biogenesis	80.12	34467	4.79	1
51	Q9JKU9	Sigma 1 receptor beta variant	Lipid biogenesis	52.54	21589	6.63	1
52	Q9R1J0	Sterol-4-alpha-carboxylate 3-dehydrogenase	Lipid biogenesis	42.14	40660	3.52	1
53	Q69ZN7	Myoferlin (Fer-1-like protein 3)	Membrane repair	152.49	236053	2.38	3
54	Q99KV1	DnaJ homolog subfamily B member 11 precursor	mRNA modification	41.04	40530	2.99	1
55	**P00762**	**Anionic trypsin-1 precursor**	Peptidase	81.51	25943	8.51	1
56	P07150	Annexin A1	Phospholipid binding/modifying	223.58	38674	7.12	3
57	**P07356**	**Annexin A2**	Phospholipid binding/modifying	934.99	38521	40.57	14
58	Q3UCL0	annexin A4	Phospholipid binding/modifying	177.72	35893	9.51	3
59	P48037	Annexin A6	Phospholipid binding/modifying	367.61	75575	14.26	8
60	P14824	Annexin A6	Phospholipid binding/modifying	359.86	75707	13.66	8
61	Q8VDP6	Phosphatidylinositol synthase	Phospholipid binding/modifying	37.88	23583	5.14	1
62	Q8R366	Immunoglobulin superfamily member 8 precursor	Protein binding	115.51	64970	3.73	2
63	**Q4FCR4**	**Intercellular adhesion molecule 1**	Protein binding	186.97	53809	8.38	3
64	P21956	Lactadherin precursor	Protein binding	47.39	51236	2.37	1
65	P70117	Pancreas cancer-associated protein 4	Protein binding	166.84	64358	6.67	3
66	P70206	Plexin-A1 precursor	Protein binding	75.36	210965	1.3	2
67	Q9WV91	Prostaglandin F2 receptor negative regulator precursor	Protein binding	91.15	98646	3.46	2
68	Q5SS40	Tyrosine 3-monooxygenase/tryptophan 5-monooxygenase activation protein	Protein binding	50.26	29155	4.91	1
69	**P62629**	**Elongation factor 1-alpha 1**	Protein biosynthesis	208.12	50082	12.97	4
70	P10630	Eukaryotic initiation factor 4A-II	Protein biosynthesis	40.71	46373	2.38	1
71	Q66H94	Peptidyl-prolyl cis-trans isomerase (PPIase)	Protein biosynthesis	44.5	63086	2.09	1
72	Q8R2Y8	Peptidyl-tRNA hydrolase 2 (PTH 2)	Protein biosynthesis	50.9	19514	7.82	1
73	Q9CY50	Translocon-associated protein subunit alpha precursor	Protein biosynthesis	44.77	32045	5.15	1
74	**Q62186**	**Translocon-associated protein subunit delta precursor**	Protein biosynthesis	58.73	18924	6.4	1
75	Q60432	150 kDa oxygen-regulated protein	Protein folding	111.56	111202	3.56	2
76	Q1PSW2	84 kDa heat shock protein	Protein folding	268.13	83230	5.29	4
77	**P24369**	**Cyclophilin B**	Protein folding	159.73	22699	17.96	3
78	**P30412**	**Cyclophilin C**	Protein folding	111.37	22780	12.08	2
79	Q8R180	ERO1-like protein alpha precursor (ERO1-Lalpha)	Protein folding	40.76	54004	2.45	1
80	Q3U5T8	t-complex protein 1	Protein folding	97.92	55438	4.17	2
81	P80318	T-complex protein 1 subunit gamma	Protein folding	82.19	60591	2	1
82	O70152	Dolichol-phosphate mannosyltransferase	Protein processing	41.77	29156	8.3	1
83	Q3UC51	dolichyl-di-phosphooligosaccharide-protein glycotransferase	Protein processing	197.03	49011	9.44	4
84	Q03145	Ephrin type-A receptor 2 precursor	Protein processing	77.94	108753	2.73	2
85	O08795	Glucosidase 2 subunit beta precursor	Protein processing	42.18	58756	1.87	1
86	P17439	Glucosylceramidase precursor	Protein processing	81.57	57585	4.59	2
87	**P57716**	**Nicastrin precursor**	Protein processing	48.9	78441	1.54	1
88	P27773	Protein disulfide-isomerase A3 precursor	Protein processing	421.29	56586	18.29	8
89	P38660	Protein disulfide-isomerase A6 precursor	Protein processing	264.65	48131	11.67	3
90	Q8R4U2	Protein disulfide-isomerase precursor (PDI)	Protein processing	516.21	56975	27.85	9
91	P52480	Pyruvate kinase isozymes M1/M2	Protein processing	257.65	57719	14.89	5
92	Q91YQ5	Ribophorin I	Protein processing	47.47	68486	1.93	1
93	Q9DBG6	Ribophorin II	Protein processing	97.57	69020	3.67	2
94	Q00993	Tyrosine-protein kinase receptor UFO precursor	Protein processing	70.28	98188	1.23	1
95	**Q6AXQ3**	**Viral oncogene yes-1 homolog 1**	Protein processing	36.55	54236	4.46	1
96	Q3TFU8	Glycoprotein 25L2 homolog	Protein transport	89.06	31239	7.42	2
97	P62835	Rap-1A precursor	Ras-related protein	240.69	20974	18.42	4
98	P80236	Ras-related C3 botulinum toxin substrate 1 (p21-Rac1)	Ras-related protein	47.41	8805	17.5	1
99	**P61226**	**Ras-related protein Rap-2b precursor**	Ras-related protein	69.2	20491	6.45	1
100	**P62071**	**Ras-related protein R-Ras2 precursor**	Ras-related protein	79.46	23385	7.55	1
101	Q61411	Transforming protein p21 (p21ras)	Ras-related protein	49.31	21335	6.22	1
102	**P17852**	**Integrin alpha-3 precursor**	Receptor	329.02	118475	6.59	5
103	P43406	Integrin alpha-V precursor	Receptor	120.21	115205	2.87	3
104	P09055	Integrin beta-1 precursor	Receptor	90.71	88173	2.5	2
105	**P61022**	**Calcium-binding protein p22**	Signalling	58.15	22287	5.94	1
106	**P62204**	**Calmodulin**	Signalling	187.09	16696	30.46	3
107	**P62874**	**G protein G(I)/G(S)/G(T) subunit beta-1**	Signalling	186.51	37222	13.02	3
108	**P62880**	**G protein G(I)/G(S)/G(T) subunit beta-2**	Signalling	151	37176	13.06	3
109	Q3HR13	Guanine nucleotide binding protein alpha inhibiting 2	Signalling	235.3	40419	14.17	4
110	Q5EAP4	Guanine nucleotide binding protein, alpha 14	Signalling	227.12	41415	12.5	4
111	P21279	Guanine nucleotide-binding protein G(q) subunit alpha	Signalling	273.99	41457	20.21	5
112	Q8R4A8	Guanine nucleotide-binding protein G(s) subunit alpha	Signalling	232.48	45621	13.04	5
113	**P62977**	**Ubiquitin**	Signalling	114.62	8560	32.47	2
114	O88736	3-keto-steroid reductase	Steroid metabolism	58.55	37293	3.54	1
115	P47953	Galectin-3	Sugar binding	50.42	25592	4.31	1
116	Q3U0D7	ADP-ribosylation factor 6	Trafficking	37.23	20069	6.04	1
117	**Q9CQW2**	**ADP-ribosylation factor-like protein 8B**	Trafficking	205.81	21525	17.44	4
118	Q9DB05	Alpha-soluble NSF attachment protein (SNAP-alpha)	Trafficking	173.24	33168	12.62	3
119	P49020	COPI-coated vesicle membrane protein p24	Trafficking	66.59	22175	6.47	1
120	P08113	Endoplasmin precursor	Trafficking	334.23	92418	5.6	5
121	P49130	LAMP-2	Trafficking	41.5	44999	2.44	1
122	O35604	Niemann-Pick C1 protein precursor	Trafficking	36.07	142795	1	1
123	Q91ZX7	Prolow-density lipoprotein receptor-related protein 1 precursor (LRP)	Trafficking	107.05	504411	0.68	2
124	O35074	Prostacyclin synthase	Trafficking	170.13	57011	7.34	3
125	Q9D0F3	Protein ERGIC-53 precursor	Trafficking	52.6	57753	2.1	1
126	**Q4FJL0**	**RAB10**	Trafficking	174.32	22559	16.1	3
127	P62492	RAB11A	Trafficking	51.03	24247	5.91	1
128	**Q91V41**	**RAB14**	Trafficking	99.99	23751	11.16	2
129	P62821	RAB1A	Trafficking	187.04	22532	19.12	3
130	**P53994**	**RAB2A**	Trafficking	263.69	23533	30.52	5
131	Q3UCX7	RAB5A	Trafficking	80.78	23585	11.68	2
132	**P61021**	**RAB5B**	Trafficking	141.52	23692	20.47	3
133	**Q3TCT9**	**RAB5C**	Trafficking	200.16	23398	23.11	4
134	**Q3U4W5**	**RAB6**	Trafficking	269.71	23531	28.64	5
135	**Q4AEF6**	**RAB7**	Trafficking	40.92	28557	3.47	1
136	**Q3UZ06**	**SEC22 vesicle trafficking protein-like 1**	Trafficking	97.82	18935	6.98	1
137	**Q8K021**	**Secretory carrier membrane protein 1**	Trafficking	40.66	38004	6.67	1
138	Q62465	Synaptic vesicle membrane protein VAT-1 homolog	Trafficking	77.87	43069	3.58	1
139	O88385	Syntaxin 12	Trafficking	55.46	31090	5.32	1
140	Q3TSL5	syntaxin 4A	Trafficking	76.27	34204	7.1	2
141	Q9JI92	Syntenin-1	Trafficking	109.68	32403	11.33	2
142	Q61235	Syntrophin-3 (SNT3)	Trafficking	162.55	56346	7.42	3
143	Q07891	Transferrin receptor protein 1 (TfR)	Trafficking	51.95	85027	1.42	1
144	**O35587**	**Transmembrane protein Tmp21 precursor**	Trafficking	152.47	24805	16.44	3
145	P63024	Vesicle-associated membrane protein 3 (VAMP-3)	Trafficking	55.54	11473	15.38	1
146	**Q791V5**	**Mitochondrial carrier homolog 2**	Transporter	71.58	33477	7.89	1
147	Q7TP91	Ab1-205	Unknown cellular process	50.91	83209	1.72	1
148	Q9D7N9	Adipocyte plasma membrane-associated protein	Unknown cellular process	94.98	46405	5.46	2
149	P20944	CD44 antigen precursor	Unknown cellular process	142.8	39750	4.99	2
150	Q9QV38	ERP6	Unknown cellular process	54.19	2112	73.68	1
151	Q8BLN5	Lanosterol synthase (EC 5.4.99.7)	Unknown cellular process	68.6	83088	1.85	1
152	Q6P7S1	N-acylsphingosine amidohydrolase 1	Unknown cellular process	150.07	44415	10.42	3
153	P97300	Neuroplastin precursor	Unknown cellular process	60.1	31258	3.52	1
154	**Q9Z239**	**Phospholemman precursor**	Unknown cellular process	38.49	10316	12.9	1
155	O55221	Putative CD98 protein	Unknown cellular process	63.88	58889	3.74	1
156	Q9JK11	Reticulon-4	Unknown cellular process	59.01	126310	1.13	1
157	Q9CWD1	similar to DB83 PROTEIN	Unknown cellular process	66.14	19857	6.67	1
158	**Q3TIP1**	**solute carrier family 3**	Unknown cellular process	55.26	10997	17.17	1
159	Q5XIK2	Thioredoxin domain-containing protein 14 precursor	Unknown cellular process	39.08	33844	3.91	1
160	Q2TBF8	Transmembrane 9 superfamily protein member 4	Unknown cellular process	41.68	74584	1.33	1
161	**Q6P791**	**UPF0404 protein C11orf59 homolog**	Unknown cellular process	116.65	17710	16.25	2

Mw: theoretical molecular weight (kDa). Cov (%), protein sequence coverage; Nb pep, number of peptides assigned. In bold are the proteins found in both DRM and DSM fractions.

**Figure 1:**
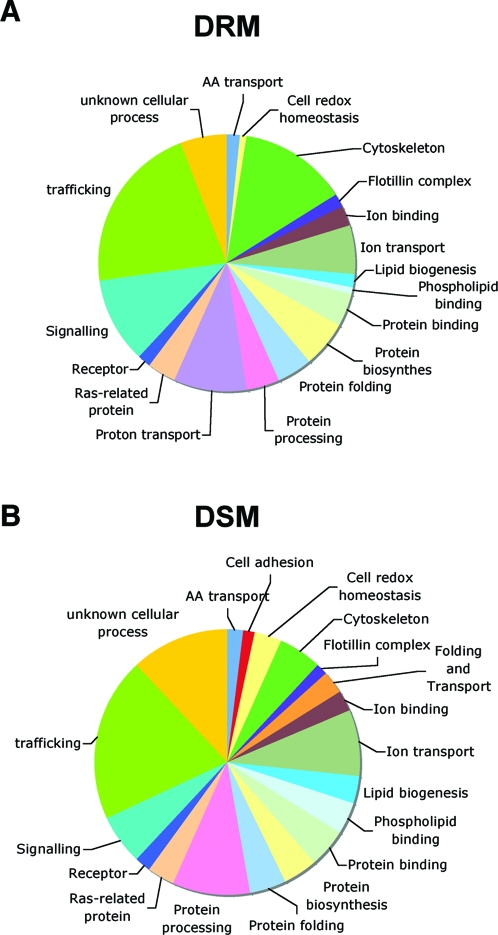
Graphic representation of the main functional categories found in the proteomic studies. A: DRM fraction, B: DSM fraction.

As illustrated in Fig. 1, proteins forming the largest group, in both fractions, were involved in trafficking, confirming that late endosomes are implicated in intensive communication with other compartments. In particular, several small GTPases of the Rab family, in addition to Rab7 well known to localize to late endosomes, were found, in agreement with other proteomic studies [40] and suggesting a more complex role/localization of Rabs than expected.

Interestingly many of the identified proteins were involved in signaling, in particular in the DRMs, such as the α and ß subunits of several G proteins, as also reported by others reported [40], [41]. The presence of these proteins in late endosomes supports the notion that endosomes are active signaling sites [42] and that cholesterol rich domains are important in organizing signaling platforms [43].

However, the most striking finding to us was the identification of all the subunits of the V-ATPase (a1, c and d from the V0 subunit and A, B2, C, D, E, F, G2 and H from the V1 subunit) exclusively in the DRM fraction. This observation suggested that the V-ATPase may localize to specific membrane domains within late endosomes and that this could be important for its function or the regulation of its activity.

There is increasing evidence that the V-ATPase can interact with numerous regulatory proteins, and thus we investigated whether some of these proteins came up in our proteomics analysis. The H-subunit, which shows homology to ß-adaptins [44], was found to interact with the m2-chain of AP2 adaptor and promotes clathrin-coated vesicle (CCV) formation [44]. Interestingly we found three of the subunits of the adaptor complex AP-2, in the late endosomal DRM fraction. Functional AP-2 complex was previously observed in lysosomes and found to support clathrin assembly and bud formation [45], [46]. We also found the small GTPase Arf6, albeit in DSM fraction. Arf6 was found to interact with the Vo c subunit and it was proposed that through this interaction, the vATPase could modulate membrane trafficking in the endosomal system [47]. In addition to mediating proton transport, it has been proposed that the Vo subunit is involved in vesicles fusion both in yeast [48] and in higher eukaryotes [49], [50]. Numerous proteins involved in membrane fusion were found in the DRM fraction including SNAP-23, syntaxin 8, VAMP-A and the v-SNARE like protein Vtil-rp1.

Finally the dissociation of the V1/vo complex, in yeast, has been shown to require an intact microtubule network [51] and well aldolase, which would act as a glucose sensor and signal for the dissociation of the V-ATPase [52]. Both tubulin and aldolase were detected in our proteomic analysis. Interestingly aldolase was only found in the DSM fraction and it is tempting to speculate that spatial segregation between the V-ATPase and aldolase is necessary to prevent uncontrolled disassembly of the complex. Clearly, non transmembrane proteins could also be removed from the detergent resistant domains during the solubilization.

### DRM association of the V-ATPase

**Figure 2:**
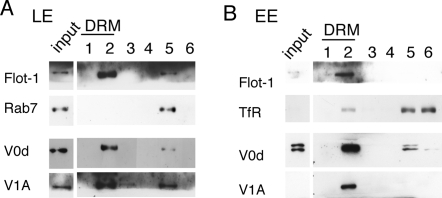
Association of V1 and V0 domains of the V-ATPase with the DRMs of early and late endosomes. Late (A) and early (B) endosomes were purified from BHK cells and the fractions were submitted to solubilization in 1% triton X-100 at 4°C. The Triton X-100 treated fractions were subsequently loaded at the bottom of an OptiPrep gradient. After centrifugation, 6 fractions were collected from the top and analyzed by SDS-PAGE followed by Western blotting to detect flotilin-1, Rab7, the vATPas subunits V0d and V1A.

To validate the DRM association of the V-ATPase revealed by the proteomic analysis, we performed a Western blot analysis probing for V-ATPase subunits of the V_1_ and V_0_ domains. Distribution of the V0 domain was monitored by following the d subunit and distribution of the V1 domain by following the A subunit. Both V_0_d and V_1_A subunits were found in the DRM fractions of both late endosomes and early endosomes (Fig. 2). Note that the total protein content of each fraction was analyzed in Fig. 2, as opposed to the same amount of protein. Fig. 2 thus shows that the majority of the V-ATPase localized to DRMs, showing a distribution similar to that of Flotillin-1, a well-established marker of DRM fractions [38].

### Effects of U18666A treatment on the acidification of late endosomes

The above experiments show that all along the endocytic pathway, from early to late endosomes, the V-ATPase exhibits a strong affinity for DRMs suggesting its preference for cholesterol rich, ordered membrane domains. To evaluate the physiological relevance of these findings, we investigated whether perturbing cholesterol rich domains in late endosomes would affect the function of the V-ATPase, by monitoring the acidity of late endosomes.

**Figure 3:**
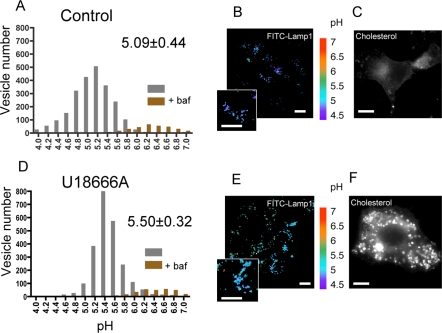
Effects of U18666A treatment on the pH at the limiting membrane (FITC-Lamp1) of late endosomes. Lamp1-FITC was internalized overnight at 37°C by control cells (A, B, C) and cells treated by U18666A (D, E, F). Typical examples of the respective labels are shown (B and E). The pH of individual organelles was measured by fluorescence ratio imaging of internalized Lamp1-FITC. The histograms show the pH distribution of 2304 and 2325 endosomes for the upper and the lower panels, respectively, with the mean±SD given in the left panels (A and D). Pseudocolor pH scale is on the side. Histograms of vesicles of cells treated with bafilomycin are shown as a control. The observed difference is significant according to a paired t-test with p<0.001. Cells were checked for their phenotype of cholesterol accumulation with filipin staining (C and F). Bars, 10 µm.

We first investigated whether extraction of cholesterol from cells using ß-methylcyclodextrin (ß-MCD) would lead to a change in late endosome acidification. Cells were treated with 10 mM ß-MCD for 55 min leading to an ≈50% decrease in total cholesterol content [53]. As illustrated in Fig. S1, ß-MCD treatment led to a significant increase in late endosomal pH, supporting the notion that alteration of cholesterol rich domains affects the function of the V-ATPase. These findings however need to be taken with a word of caution, since we have no evidence that extraction of cholesterol from the plasma membrane actually leads to a significant reduction of cholesterol in late endosomes. Indeed when purifying late endosomes from ß-MCD treated cells, we could not detect a significant change in cholesterol, as analyzed by thin layer chromatography, a technique that might not be sensitive enough to detect small changes (10–20%). We therefore decided to induce perturbation of late endosomal membrane domains by treating cells with the negatively charged amine 3beta-(2-diethylaminoethoxy)-androstenone HCl (U18666A) [54], which leads to the accumulation of cholesterol in late endosomes, through unknown mechanisms, and to alterations in the membrane dynamics of this compartment [34]. Treatment for 18 hrs with U18666A mimics the Niemann Pick type C phenotype [34], [54], [55] showing a drastic cholesterol accumulation in late endosomes (Fig. 3CF and 4CF).

**Figure 4:**
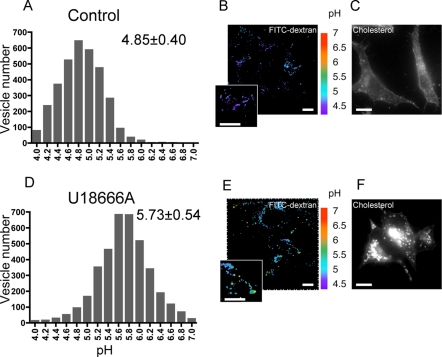
Effects of U18666A treatment on the pH of late endosomal lumen (FITC-dextran). FITC-dextran was internalized for 15 minutes and chased for 40 minutes at 37°C in control cells (A, B, C) and cells treated by U18666A (D, E, F) to allow it to reach late endosomes. Typical examples of the respective labels are shown (B and E). The pH of individual organelles was measured by fluorescence ratio imaging of internalized FITC-Dextran. Pseudocolor pH scale is on the side. The histograms show the pH distribution of 3346 and 3880 endosomes for the upper and the lower panels, respectively, with the mean±SD given in the left panels (A and D). The observed difference is significant according to a paired t-test with p<0.001. Cells were checked for their phenotype of cholesterol accumulation with filipin staining (C and F). Bars, 10 µm.

The pH measurements were performed by fluorescence ratio imaging, using the pH sensitive florescent probe FITC (fluorescein isothiocyanate) [56]. We used two different probes: FITC coupled to dextran, which was endocytosed by fluid phase and thus provides information on the bulk of the endosomal lumen, and FITC coupled to a monoclonal antibody, 4A1, against the luminal domain of the hamster late endosomal protein Lamp1. Lamp1 localizes specifically to the limiting membrane of late endosomes [57] –this organelle being multivesicular [23]–, and thus FITC anti-lamp1 allows the measurement of the pH in the vicinity of the limiting membrane. We find that the 4A1 anti-Lamp1 antibody internalized overnight (Fig. 3B, 3E), reaches late endosomes where it binds to its antigen (co-localizing with the late endosomal lipid LBPA, not shown) and is not degraded, in agreement with observations by others [58], [59]. In contrast, a none specific antibody endocytosed at the same concentration was degraded in late endosomes/lysosomes, as documented by others [59]. The fluid phase fluorescent probe, FITC-Dextran was internalized for 15′ and chased for 40′ in order to reach late endosomes (Fig. 4B, 4E). Importantly we have previously shown that U18666A treatment does not prevent transport to late endosomes [34]. Series of images were taken at both 440 nm and 490 nm, prior to calibration with different pH solutions, which allows the translation of ratio values into pH values. As illustrated by the pseudocolor images (Fig. 3B, 4B) and quantified in the distribution histograms (Fig. 3A, 4A), the pH of late endosomes in control cells varied between 4.85±0.40 and 5.09±0.44 depending on the probe used, a pH that was neutralized upon treatment with the V-ATPase specific inhibitor bafilomycin (Fig. 3A). Late endosomes of U18666A cells were however 0.41 (using anti-lamp1 FITC) to 0.88 (using FITC dextran) pH units less acidic than late endosomes from control cells, yet pH was still sensitive to bafilomycin, suggesting that acidity was still mainly due to the V-ATPase.

**Figure 5:**
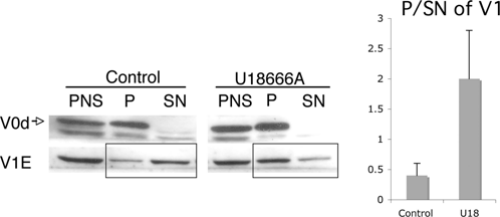
Increase of V1 association to membrane upon U18666A treatment. PNS was submitted to high speed spinning and pellet (P) and supernatant (SN) were loaded on a gel. Western blotting was revealed with V0d and V1E subunit antibody. Quantification reveals that upon U18666A treatment, V1E is 4-fold more associated with the membrane (pellet) than in control cells.

We next analyzed the effect of U18666A biochemically, monitoring the association of the V-ATPase subunits with membranes. Post nuclear supernatant (PNS) from control and U18666A treated BHK cells were submitted to high-speed centrifugation to separate the membranes (P: pellet) from the cytosol (SN: supernatant). As expected for a V_0_ domain subunit, V_0_d was entirely membranes associated, irrespective of U18666A treatment. In contrast, the majority of V_1_E was in the cytosolic fraction of control cells with only a minor fraction associated with membranes. Interestingly, this distribution was reversed upon U18666A treatment, leading a two-fold increase in the membrane to cytosol ratio of the V_1_E subunit (Fig. 5). This result indicates that the lipid composition of the membrane, and possibly the degree of order of the membrane, affects the V1-V0 association/dissociation of the V-ATPase. The observations are compatible with a higher V1 off rate in a more fluid membrane. The transport of protons by the V-ATPase requires the association of the V1 sector with the V0. Therefore an increased association of V1 with membranes as observed upon U18666A would a priori be expected to lead to an increased activity and thus more acidic endosomes. Yet U18666A led to a decrease in acidity. Together these observations suggest that U18666A altered the dynamics of association/dissociation, which appear to be essential for proper V-ATPase function, leading to a locked configuration of the V-ATPase.

### Increase of the V_1_/V_0_ ratio along the endocytic pathway

**Figure 6:**
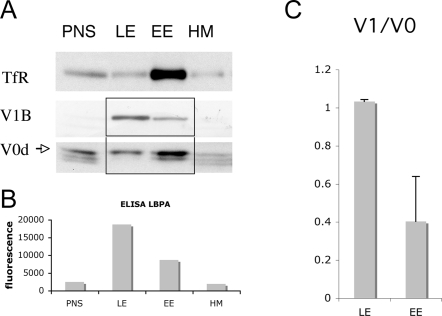
Increased assembly of the two sectors of the V-ATPase in late endosome. Subcellular fractionation of postnuclear supernatants (PNS) was performed to separate early (EE) from late endosomes (LE) and heavy membranes (HM) A: 20 µg of protein from each fraction were separated in 12.5% SDS-PAGE and blotted for the presence of TfR (early endosomal marker), V0d and V1B. B: The late endosomal lipid LBPA was used to follow the distribution of late endosomes on the gradient. An equal amount of protein was used for LBPA detection by ELISA with the LBPA antibody (6C4). C: Western blot was quantified using a Phosphoimager (Bio-Rad Laboratories) and the ratio V1/V0 was plotted. Note that the ratio LE/EE is higher for the V1B subunit than for the V0d subunit suggesting a higher assembly of the V-ATPase in the late endosomes compared to early endosomes.

The above observation suggests that the membrane environment influences the activity of the V-ATPase. This does not exclude other mechanisms of regulation and in particular we found that the V-ATPase was associated with DRMs in both early and late endosomes, rendering it unlikely that membrane localization is the sole responsible for the different pH in early and late endosomes. We therefore investigate whether association/dissociation of the V1 domain form the V0 domain, the regulatory mechanism found in yeast, could contribute to the increase in acidity along the endocytic pathway. Early and late endosomes fractions, enriched in the transferrin receptor and the late endosomal lipid lysobisphosphatidic acid (LBPA, used a marker of late endosomes) respectively (Fig. 6A and 6B), were obtained by differential centrifugation from BHK cells and separated from heavy membranes containing the endoplasmic reticulum and the Golgi (Fig. 6). As expected, the V_0_ and V_1_ subunits, revealed with anti- V_0_d and anti-V_1_B respectively, were detected in both early and late endosomes (Fig. 6A). As observed through the analysis of multiple experiments, the enrichment of the V0 subunit d was quite similar in early and late endosomes indicating that the density of the proton translocator subunit was the same in both compartments (not shown). What was strikingly different between early and late endosomes was however the relative abundance of the V1 domain, as witness by following the B (Fig. 6A), the A or E subunits (data not shown): the V1/V0 ratio was always higher in late than in early endosomes, as quantified from several experiments in Fig. 6B (n = 3, note that the ratios only have a relative meaning and do not provide stochiometric information because the antibodies against the two subunits are by definition different). The higher V1/V0 ratio in late vs. early endosomes correlates with the higher acidity of this compartment, suggesting that an increase in V-ATPase activity through an increase in V1-V0 association is the underlying mechanism and that this mechanism of regulation is not restricted to yeast [14], [60] and maturing dendritic cells [16].

## Discussion

Endosomes are the most acidic organelles in mammalian cells, yet little is known about the exact mechanisms that regulate their pH and how acidity increases along the endocytic pathway. Although the V-ATPase is the main contributor to this acidity, how its activity is regulated is to a large extent unknown. The main mechanism described to date is the reversible dissociation of the V_0_ and V_1_ domains of the V-ATPase. It was first discovered in yeasts and insects. In these organisms however, dissociation appears as a survival mechanism to conserve the ATP stock upon conditions of energy limitation by glucose deprivation or starvation [7], [14], [15]. The known glucose-induced signaling pathways do not seem to be involved since mutants in the ras-cyclic AMP pathway or in the protein kinase C pathway, continue to assemble in the presence of glucose [7], [61]. Regulation by reversible dissociation has also been described in specialized mammalian cells, such as renal epithelial cells, where reversible dissociation is, as in yeast, coupled to glucose levels [62], [63]. The only situation in which changes in V1-V0 association were aimed at controlling endosomal pH was during maturation of dendritic cells, where increased acidification was shown to increase the activity of hydrolytic enzymes and thus the efficiency of antigen processing [16]. Our work suggests that, as in maturing dendritic cells, increased assembly of the V-ATPase sectors along the endocytic pathway of fibroblast-like cells contributes to increasing the acidity of the organelles.

Changes in V1-V0 association raise the questions of the mechanisms that regulate this process. Our striking finding that all the subunits of the V-ATPase localized to the DRM fraction and that treatment of cells with U18666A, which affects late endosomal cholesterol levels, lead to an increase in V1-V0 association, together point to a role of the membrane composition in controlling the rate of V1-V0 association-dissociation. This could be either through the direct effect of specific lipids on the V-ATPase mediated by a lipid-protein interaction, or/and through an effect of the membrane fluidity. High fluidity, as possibly found at the plasma membrane, would lead to V1 dissociation and a drop in V-ATPase mediated acidification. Similarly low membrane fluidity, as promoted by U18666A dependent cholesterol accumulation, would lead to a too strong V1-V0 interaction preventing proper function of the proton pump, and thus again reduction in activity. The effect of U18666A is reminiscent of the marine-derived V-ATPase inhibitor salicylihalamide A. This metabolite binds to the Vo complex, as do Bafilomycin A or concanamycin, but to a different site. More strikingly, salicylihalamide A triggers a dramatic redistribution of the V1 complex from the cytoplasm to the endosomes, a redistribution not observed with the other inhibitors [64]. The paradoxical effects of U18666A and salicylihalamide A suggest that excessive stabilization of the V1-V0 complexes is detrimental to the pumps activity.

The hypothesis that raft-like domains are involved in regulating the activity of the mammalian V-ATPase is further supported by findings by others, in yeast, that sphingomyelin [20], and possibly ceramide [65] modulate the function of the V-ATPase. Interestingly, ceramide was also found to be required for oligomerization of the plasma membrane proton ATPase in yeast [66].

We wish to mention that the V-ATPase has previously been found in a raft fraction of the plasma membrane of neurons [67]. However only components of the V0 domain were identified. In contrast, we here found all eight V1 subunits (table 1). Yoshinaka et al. [43] hypothesize that the V-ATPase may disassemble during sample preparation or that the V0 sector selectively associates with rafts. Our results show that both V1 and V0 sectors can be recovered in the DRM fraction. The apparent discrepancy between the two studies could be due to the fact that plasma membrane fractions were analyzed in the first study and that the V-ATPase is mostly disassembled at that cellular site as opposed to endosomes studied here. This interpretation is supported by our finding that assembly of V1 and V0 increases along the endocytic pathway and would thus be lowest at the cell surface.

The here identified effect of membrane composition on the activity of the V-ATPase suggests that diseases that affect lipid distribution or composition, in particular in the endocytic pathway, may lead to alterations in endosomal pH. It will therefore be of interest to determine if endosomal pH is affected in cells from patients suffering from lipid storage diseases such as Niemann Pick type C and how this affects endosomal function.

## Materials and Methods

### Cell, reagents and drug treatment

Monolayers of Baby hamster kidney (BHK) cells were grown and maintained as described previously [68], [69]. The monoclonal antibodies against LBPA (6C4) and Lamp 1 (4A1) have been described [58], [70]. Monoclonal anti-transferrin receptor antibodies were from Zymed Laboratories Inc. Rabbit polyclonal anti-flotillin antibodies were produced by our laboratory [25]. Polyclonal antibody against the 39-kDa subunit of the V-ATPase (V0d) was previously described [71]. Rabbit polyclonal antibodies against V1 subunit A and V1 subunit E were kind gifts from I. Schultz and antibodies against V1 subunit B were produced by Eurogentech. Rhodamine-dextran (10,000 Da) was from Molecular Probes. For pH measurement, Lamp1 antibody was labeled with FITC (Fluo reporter labeling kit) from Molecular probes. Cells were treated with 3beta-(2-diethylaminoethoxy)-androsterone HCl (U18666A) as described [34]. Briefly, the cell culture medium was removed 4 hours after plating and fresh medium containing U18666A at 3 µg/ml was added for 18 hours.

### Subcellular fractionation and Immunoblotting

Early and late endosomal fractions were prepared as described [32], [34]. Briefly, BHK cells were homogenized, a post nuclear supernatant (PNS) was prepared, adjusted to 40.6% sucrose, loaded on the bottom of a SW41 tube and overlaid sequentially with 35%, 25% and 8.5% sucrose solution in 3mM imidazole, pH 7.4. The gradient was centrifuged for 90 minutes at 35000 rpm. Heavy membranes, early and late endosomal fractions were collected at the 40.6/35%, 35/25% and 25/8.5% interfaces respectively. Proteins were separated by SDS-PAGE using 12.5% acrylamide gels and transferred onto a nitrocellulose membrane. Western blots were revealed with SuperSignal Chemiluminescence (Pierce) and quantified by densitometry.

### Isolation of DRMs from early and late endosomal fraction

DRMs were prepared from late endosomes as described [25]. Early or late endosomes were diluted four times, sedimented by centrifugation (TLS55 Beckman Rotor, 30 min, 55000 rpm) and resuspended in 200 µl of lysis buffer (25 mM Tris-HCl pH 7.4, 150 mM NaCl, 5 mM EDTA) in the presence of Complete, a cocktail of protease inhibitors (Roche) and 1% Triton X-100. After 20 min of incubation at 4°C, the lysat was adjusted to 40% OptiPrep (Nycodenz), overlaid with 30% and 0% OptiPrep cushions and centrifuged for two hours at 55000 rpm (4°C) using a TSL55 rotor. Six fractions were collected and precipitated with 6% trichloroacetic acid in the presence of sodium deoxycholate as a carrier.

### Sample preparation for mass spectrometry analysis

DRMs and DSMs fractions were prepared from late endosomes (200 µg) as explained in precedent paragraph. 30 µg proteins from recovered DRMs and DSMs fractions were precipitated using methanol/chloroform. Proteins were then run on 2 cm in one-dimensional SDS-PAGE (15% acrylamide gel, 0.75 mm thick). Protein bands (12, and 14 for DRMs and soluble fraction, respectively) were excised and destained by repeated cycles of incubation in 25mM NH_4_HCO_3_ for 15 min and then with 50% (v/v) ACN in the same buffer (25mM NH_4_HCO_3_) for 15 min. After drying by vacuum centrifugation, the gel pieces were incubated with an oxidizing solution (7% H_2_O_2_) for 15 min [72]. Gel pieces were then washed in HPLC grade water (Sigma-Aldrich) for 15 min before being dehydrated with 100% ACN. In-gel digestion was performed (0.5 µg trypsin/band; sequencing grade modified trypsin, Promega) in 25mM NH_4_HCO_3_ overnight at 37°C. Peptides were extracted from the gel using passive diffusion in the following solutions: 50% ACN, then 5% formic acid, and finally 100% ACN. The extracts were dried by vacuum centrifugation and peptides were resolubilized in 5% ACN, 0.2% formic acid.

### MS/MS analysis

The different sample fractions were injected into a CapLC nanoLC system (Waters) and first preconcentrated on a 300 µm×5 mm precolumn (PepMap C18; Dionex). The peptides were then eluted onto a C18 column (75 µm×150 mm; Dionex). Chromatographic separation used a gradient transition from solution A (2% acetonitrile, 98% water and 0.1% formic acid) to solution B (80% acetonitrile, 20% water and 0.08% formic acid) over 60 min at a flow rate of 200 nl/min. The LC system was directly coupled to a mass spectrometer (QTOF; Waters). MS and MS/MS data were acquired and processed automatically using MassLynx software (Waters). Database searching was performed using MASCOT 2.2 software (Matrix Science) using SwissProt_Trembl as the database and Rodent as the taxonomy. Variable modifications accorded were: Acetyl N-terminal of protein, simple and dioxidation of Methionine and cysteic acid on cysteine. Precisions on both MS and MS/MS data were set to 0.3 Da. The .dat files obtained through Mascot were further filtered using an “in-house” parsing solution (Irma, to be published: *Bioinformatics* Application Notes) build from Mascot Parser. The two sets of data were checked for false positive but none were found. Peptides whose score were > =  to query identity threshold (p<0.05) and rank < = 1 were marked as significant through a first filtering and at final, a manual validation was done on proteins containing only one or two peptides. The peptide sequences were considered as validated if they contained at least 3 consecutives Y or B ions with a S/N threshold >3. Classical MS contaminants such as trypsin and keratin proteins were removed manually.

### Fluorescence microscopy

Cells grown on cover slips were fixed with 3% paraformaldehyde for 20 min at room temperature and saturated with 10% phosphate-buffered saline-fetal calf serum (PBS-FCS) for 20 min. Cholesterol was labeled with 50 µg/ml Filipin in 10% PBS-FCS.

### pH measurements

BHK cells grown on glass cover slips, were incubated with a FITC conjugated Lamp1 antibody overnight at 37°C or a FITC dextran for 15 minutes at 37°C and washed for 40 minutes. Late endosomal pH was measured by ratio fluorescence imaging as described [73], [74] with the use of a Nipkow dual spinning disk confocal laser imaging system with a QLC module (Visitron systems GmbH, Switzerland). Cover slips were inserted into a perfusion chamber (Medical Systems, Green- vale, NY) at 37°C in 1 ml of IM medium and imaged with a video/CCD camera controlled by MetaMorph/Metafluor imaging software. Images were acquired for 500 ms at two different wavelengths, using the two lasers 490 and 440 nm. Calibration and image processing were performed as described previously [73]. Data were graphed using the Prism software.

## Supporting Information

Figure S1 Effects of β−MCD treatment on the pH of late endosomal lumen (FITC-dextran). FITC-dextran was internalized for 15 minutes and chased for 40 minutes at 37°C in control cells (A) and cells treated by β−MCD (B) to allow it to reach late endosomes. The histograms show the pH distribution of 3700 and 2194 endosomes for the upper and the lower panels. Values represent the mean±SD. The observed difference is significant according to a paired t-test with p<0.001.
(1.24 MB TIF)
