# Supplementary figures and images for: Regulation of the V-ATPase along the Endocytic Pathway Occurs through Reversible Subunit Association and Membrane Localization
Source: PLoS One. 2008 Jul 23;3(7):e2758. doi: 10.1371/journal.pone.0002758 (PMC2447177; doi:10.1371/journal.pone.0002758)

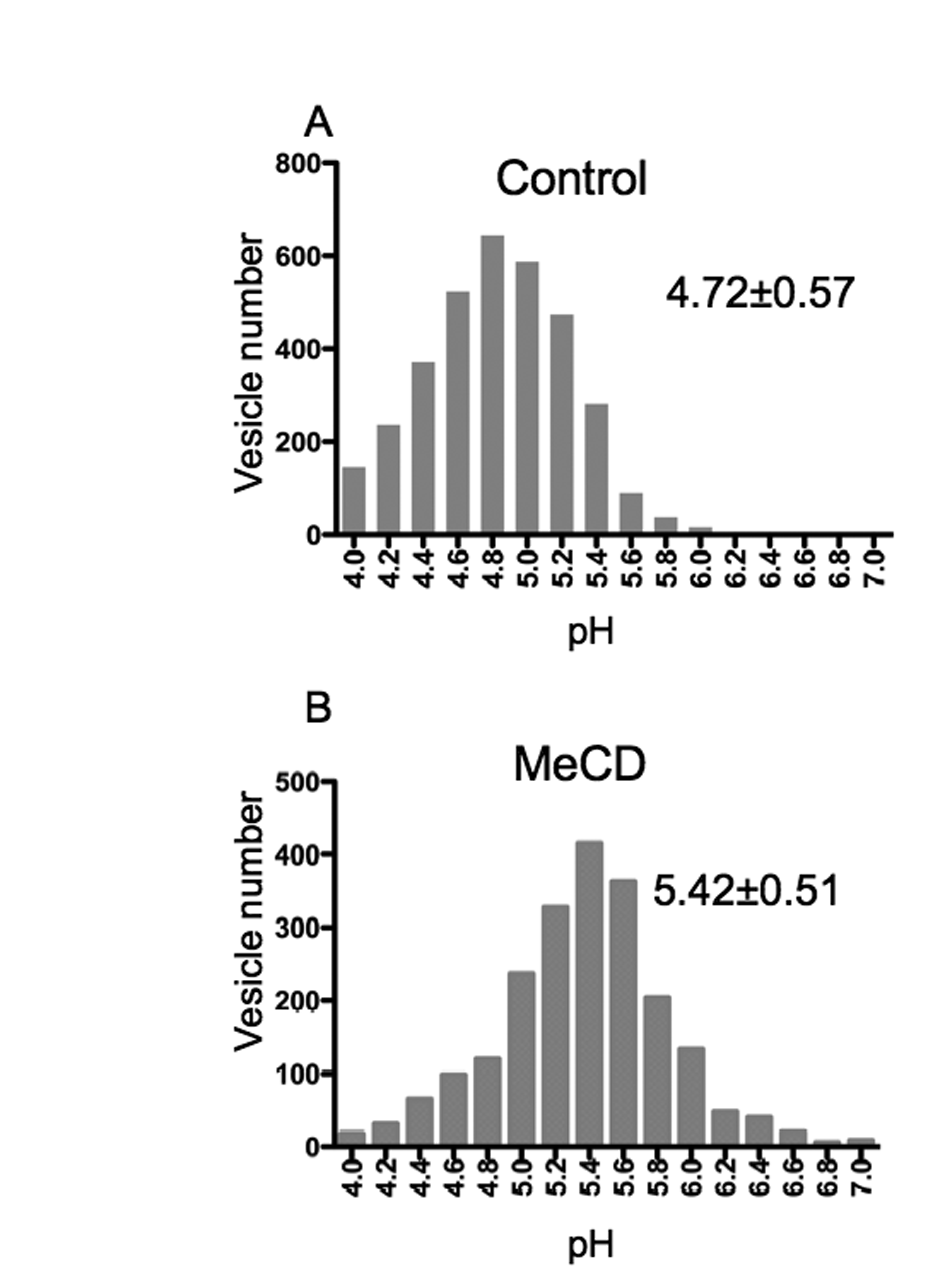

Supplement: Figure S1 — Effects of β−MCD treatment on the pH of late endosomal lumen (FITC-dextran). FITC-dextran was internalized for 15 minutes and chased for 40 minutes at 37°C in control cells (A) and cells treated by β−MCD (B) to allow it to reach late endosomes. The histograms show the pH distribution of 3700 and 2194 endosomes for the upper and the lower panels. Values represent the mean±SD. The observed difference is significant according to a paired t-test with p<0.001. (1.24 MB TIF) [file pone.0002758.s001.tif]
